# Large Area Transfer of Bismuth‐Based Layered Oxide Thin Films Using a Flexible Polymer Transfer Method

**DOI:** 10.1002/smsc.202400114

**Published:** 2024-06-30

**Authors:** James P. Barnard, Jianan Shen, Benson Kunhung Tsai, Yizhi Zhang, Max R. Chhabra, Ke Xu, Xinghang Zhang, Raktim Sarma, Aleem Siddiqui, Haiyan Wang

**Affiliations:** ^1^ School of Materials Engineering Purdue University West Lafayette IN 47907 USA; ^2^ Nanostructure Physics Sandia National Laboratories Albuquerque NM 87185 USA; ^3^ Center for Integrated Nanotechnologies Sandia National Laboratories Albuquerque NM 87185 USA; ^4^ Biological and Chemical Sensors Sandia National Laboratories Albuquerque NM 87185 USA; ^5^ School of Electrical and Computer Engineering Purdue University West Lafayette IN 47907 USA

**Keywords:** Bi_3_Fe_2_Mn_2_O_*x*_, film transfer, layered supercell, Sr_3_Al_2_O_6_, strained growth

## Abstract

Magnetic and ferroelectric oxide thin films have long been studied for their applications in electronics, optics, and sensors. The properties of these oxide thin films are highly dependent on the film growth quality and conditions. To maximize the film quality, epitaxial oxide thin films are frequently grown on single‐crystal oxide substrates such as strontium titanate (SrTiO_3_) and lanthanum aluminate (LaAlO_3_) to satisfy lattice matching and minimize defect formation. However, these single‐crystal oxide substrates cannot readily be used in practical applications due to their high cost, limited availability, and small wafer sizes. One leading solution to this challenge is film transfer. In this demonstration, a material from a new class of multiferroic oxides is selected, namely bismuth‐based layered oxides, for the transfer. A water‐soluble sacrificial layer of Sr_3_Al_2_O_6_ is inserted between the oxide substrate and the film, enabling the release of the film from the original substrate onto a polymer support layer. The films are transferred onto new substrates of silicon and lithium niobate (LiNbO_3_) and the polymer layer is removed. These substrates allow for the future design of electronic and optical devices as well as sensors using this new group of multiferroic layered oxide films.

## Graphical abstract

An epitaxial bismuth‐based layered oxide thin film is transferred from the original substrate to a new substrate. A water‐soluble oxide layer is used to release the film from the original substrate. A polymer support is then used to transport the film to the new substrate, where the film is reattached.

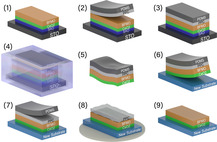

## 1 Introduction

For decades of work on thin film growth, epitaxy has been a significant challenge. Multiple factors, such as lattice parameter, crystal structure, and chemical compatibility, have been found to dictate the growth of single‐crystal‐like epitaxial films.^[^ ^1^ ^]^ This ultimately has the effect of limiting the substrates on which a given film can be deposited. When other substrates are needed for specific applications, the insertion of buffer layers has been demonstrated as one possible solution to maintaining epitaxy. Complex buffer layer stacks can be developed to allow epitaxial growth of films on many different substrates, but this process is time consuming, difficult, and must be repeated for every new substrate, as reported in previous works.^[^ ^2^ ^]^ However, new methods have revolutionized the field by allowing these challenges to be bypassed almost entirely through thin film transfer. In this process, the film is grown on the original substrate in such a way that it can be removed to become a free‐standing film and then be reattached to a new substrate. This is groundbreaking as this method allows for the quick transfer of films onto any substrate. Therefore, new applications can be explored without worrying about the challenges of epitaxy for every substrate.

There are two common methods to enable removal of the film from the substrate. The first method relies on the insertion of a 2D Van der Waals (VdW) layer such as graphene or hexagonal boron nitride (hBN) between the film and substrate. This allows the film to be “peeled” away from the substrate along the VdW layer as no physical bonding is present in the out‐of‐plane direction to secure the film.^[^ ^3^, ^4^, ^5^, ^6^ ^]^ The second method is to insert a dissolvable sacrificial layer between the film and substrate, such as Sr_3_Al_2_O_6_ (SAO), soluble in water, or La_0.7_Sr_0.3_MnO_3_ (LSMO), soluble in a solution of hydrochloric acid (HCl) and potassium iodide (KI), allowing for release of the film through dissolution.^[^ ^6^, ^7^, ^8^, ^9^, ^10^, ^11^, ^12^ ^]^ In either case the film is released from the original substrate while theoretically maintaining the as‐grown crystal structure and quality. As the film is released, it is typically supported by a flexible polymer layer, such as polycarbonate (PC), polyethylene terephthalate (PET), polypropylene carbonate (PPC), or poly(dimethylsiloxane) (PDMS) to prevent mechanical damage to the film while in this freestanding state.^[^ ^3^, ^4^, ^5^, ^7^, ^8^, ^9^ ^]^ In some cases, the film was left on this polymer support and further characterization was conducted on the free‐standing film to understand how the release process impacts the film properties and how the film on a flexible polymer may be useful, such as for flexible electronics for healthcare and consumer electronics.^[^ ^13^, ^14^, ^15^, ^16^, ^17^ ^]^ While films can be grown directly on flexible 2D substrates such as muscovite mica, epitaxial growth is limited due to challenges with VdW epitaxy.^[^ ^18^, ^19^, ^20^, ^21^ ^]^ The ability to adapt any film in a flexible device has apparent advantages. However, in many cases the process does not end here, instead continuing with adhering the film onto a new surface, which is chosen based on the targeted applications. For example, films have been transferred from SrTiO_3_ (STO), a common oxide growth substrate, onto silicon (Si) for semiconductor applications where the electrical and magnetic properties of the film were useful.^[^ ^7^ ^]^ In contrast, the new surface does not have to be a bare substrate. In many cases the film is released onto a partial film stack to construct a device, such as creating a thin film capacitor from BaTiO_3_ and SrRuO_3_ by transferring one layer at a time to complete the “sandwich” structure.^[^ ^9^ ^]^

Upon reviewing the current state‐of‐the‐art in thin film transfer, there is a glaring lack of work on the transfer of highly strained films. Film strain is typically introduced through lattice mismatch between a film and the substrate, leading to misfit strain. While it has been repeatedly shown, as discussed previously, that it is possible to transfer unstrained films using this technique, strained films introduce new challenges as the residual stress in the film is relaxed when the substrate clamping effect is lost upon removal from the original substrate. Limited work has been performed to take advantage of low misfit strain (around 1%) to tune the properties of transferred films but work on the transfer of highly strained films has hardly scratched the surface of what is possible.^[^ ^9^ ^]^ It is expected that curling, wrinkling, and cracking of the film will occur when the misfit strain is relaxed.

Highly strained oxide films present novel physical properties, such as highly coupled ferromagnetic and ferroelectric properties in multiferroic thin films.^[^ ^22^, ^23^, ^24^, ^25^ ^]^ Examples of popular multiferroic systems include BiFeO_3_ (BFO) and BiMnO_3_ (BMO).^[^ ^26^, ^27^, ^28^ ^]^ A recently discovered addition to the family of bismuth‐based multiferroic films is the Bi_3_Fe_2_Mn_2_O_*x*_ (BFMO) system.^[^ ^2^, ^29^, ^30^, ^31^, ^32^, ^33^, ^34^, ^35^, ^36^, ^37^ ^]^ The BFMO thin film material has been reported to grow in two distinct phases with very different structures.^[^ ^30^, ^36^, ^37^ ^]^ The first is the epitaxial pseudocubic phase, which has less desirable ferroelectric and magnetic properties. This phase is grown under low strain conditions (<1%).^[^ ^36^ ^]^ The second is much more interesting and is called the “layered supercell” (LSC) phase. In this phase, monolayers of Bi_2_O_2_ and FeO_6_/MnO_6_ self‐assemble during growth, resulting in an anisotropic structure and novel properties. The LSC phase has been reported to have superior ferroelectric and magnetic properties, making it the focus of this work.^[^ ^37^ ^]^ Previous works show that the LSC phase of BFMO only forms when the misfit strain is high (≈ 4%), making it more challenging to grow than the pseudocubic phase.^[^ ^36^ ^]^ Therefore the issue of strain first must be addressed before considering the possibility of transferring this highly strained BFMO material.

In this work, the successful transfer of BFMO thin films onto various substrates is demonstrated using a water‐soluble SAO sacrificial buffer layer on STO substrates. The water‐soluble SAO layer enables film liftoff as has been demonstrated in other works.^[^ ^7^, ^8^, ^10^, ^11^, ^12^, ^38^, ^39^ ^]^ The SAO sacrificial layer (*a*_SAO_ = 15.844 Å) can grow epitaxially on STO (*a*_STO_ = 3.905 Å) with a 4:1 unit cell matching (*a*_SAO_/4 = 15.844 Å).^[^ ^7^ ^]^ After the SAO layer is deposited, a CeO_2_ buffer is used to promote the growth of the subsequent BFMO LSC layer. This CeO_2_ film is important for LSC systems as it has been shown to promote the growth of the desired LSC phase instead of the more easily grown pseudocubic phase, which has inferior properties.^[^ ^2^, ^31^, ^32^, ^34^, ^35^, ^36^, ^37^ ^]^ There are two reasons for this seeding effect. First, there is a very similar zigzag structure present in both CeO_2_ and LSC phases. Second, CeO_2_ ($a_{{\text{CeO}}_{2}}$ = 5.441 Å) can form a 45‐degree rotation ($a_{{\text{CeO}}_{2}}$ $/\sqrt{2}$ = 3.826 Å) relative to the BFMO LSC phase and epitaxially match very well with the Bi–Bi spacing in the BFMO LSC lattice (*a*_BFMO_ = 4.000 Å).^[^ ^32^ ^]^ As for the substrate side, the CeO_2_ buffer layer can also form a 45‐degree in‐plane rotation on STO (3.905–3.826 Å).^[^ ^32^ ^]^ Therefore, in a common BFMO LSC sample, CeO_2_ will be used as a buffer layer on an STO substrate. In this case, the CeO_2_ buffer layer is being grown on the SAO sacrificial layer rather than on the STO substrate directly, yet the CeO_2_ buffer layer can still be used because of the combination of 4:1 domain matching epitaxy and the 45‐degree rotation matching epitaxy (4 × $a_{{\text{CeO}}_{2}}$ $/\sqrt{2}$ = 15.304–15.844 Å). All the epitaxial relationships described here are simplified to a single image in Figure S1, Supporting Information. Microstructural, property, and macroscale quality analyses are performed on the films before, during and after the transfer process. The targeted substrates for the post‐transfer films include a reference STO substrate, to isolate the effects of the transfer process; lithium niobate (LNO), a common substrate for optical and acoustic applications;^[^ ^40^, ^41^, ^42^, ^43^ ^]^ and silicon, a dominant substrate for electronic devices.

## 2 Results and Discussion

**Figure 1:**
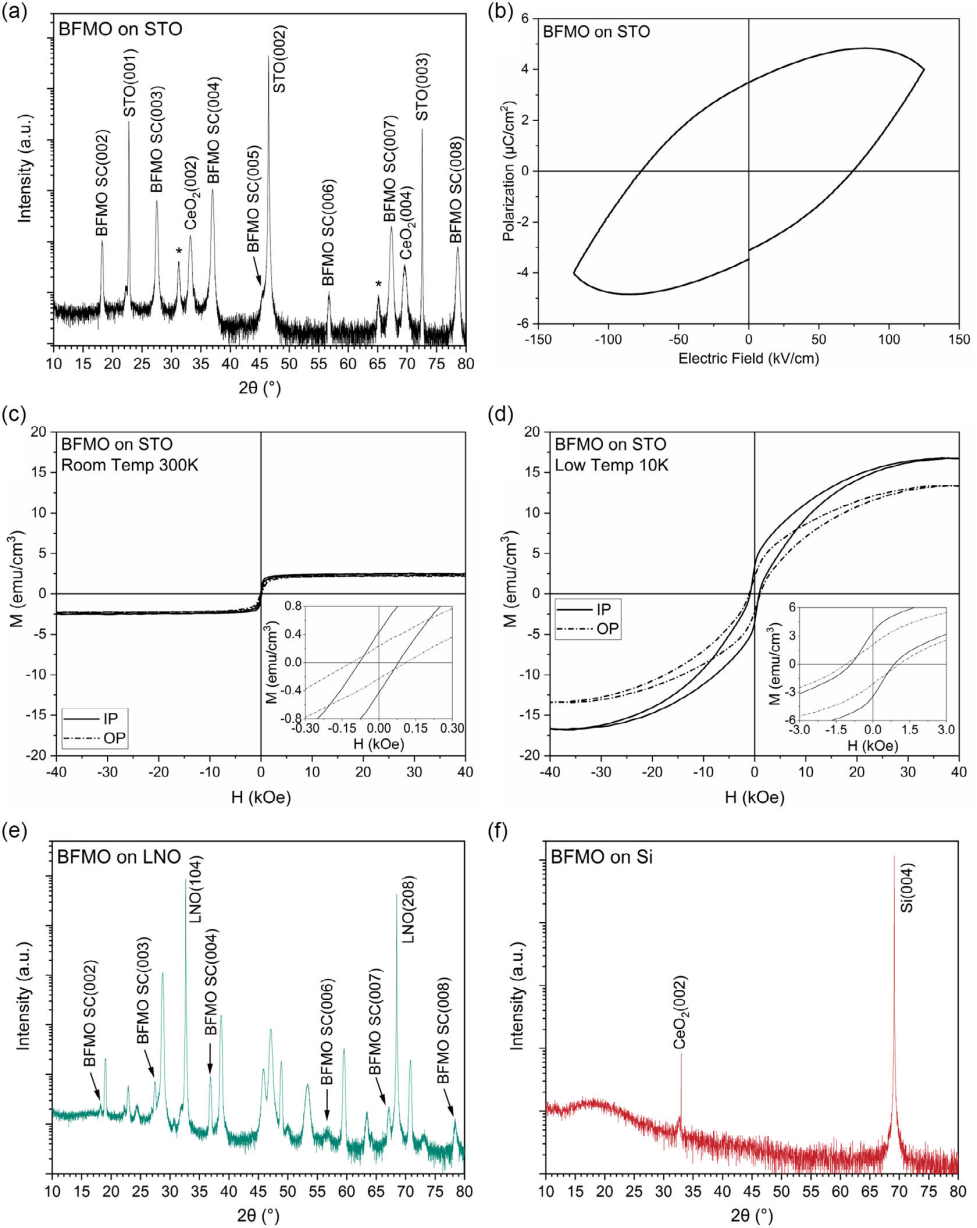
Property and microstructural analysis of direct‐grown BFMO films on various substrates. a) XRD of BFMO grown on an STO substrate with a CeO_2_ buffer layer. Peaks marked with the “*” symbol are attributed to a highly strained interlayer at the substrate interface and small grains of varied compositions. b) Ferroelectric P–E loop of BFMO on STO showing leaky ferroelectric properties. Magnetic M‐H loops of BFMO on STO at c) 300 K and d) 10 K. XRD of BFMO on e) LNO and f) Si substrates showing poor growth quality when depositing directly onto these substrates.

Before inserting the SAO water‐soluble layer, BFMO films were characterized to establish a baseline of the film epitaxial quality and multiferroic properties. The as‐deposited LSC BFMO film was first analyzed with X‐ray diffraction (XRD) to understand the single‐crystal quality. The results, shown in **Figure** **1**a, display excellent phase purity of the LSC phase and epitaxial growth with the CeO_2_ buffer layer on the STO substrate. While there are two unidentified peaks, the intensity of these peaks is relatively low. The data is plotted on a logarithmic scale and these peaks are 1–2 orders of magnitude lower than the identified CeO_2_ and BFMO LSC peaks. Similar peaks have been reported in most other BFMO LSC works and have been linked to either the highly‐strained interlayer that forms at the interface of the BFMO LSC or small grains of varied compositions.^[^ ^2^, ^30^, ^31^, ^32^, ^34^, ^35^, ^36^, ^37^ ^]^ These previous works have shown, via TEM and other techniques, that the films are still of very high epitaxial quality and that the properties are not significantly impacted.

Study of the ferroelectric properties yielded the polarization–electric field (P–E) loop shown in Figure 1b. The BFMO LSC has previously been shown to be a very leaky, weak ferroelectric through P–E loops and piezoelectric force microscopy (PFM) switching data.^[^ ^2^, ^33^, ^35^, ^36^, ^37^ ^]^ In a previous work, an FeFET was fabricated with the BFMO LSC material to take advantage of the ferroelectric properties to demonstrate the applicability of this film.^[^ ^2^ ^]^ It is possible that approaches such as increasing the film thickness, tuning the composition, or reducing the defect density could lower the leakage and bring out superior ferroelectric properties in BFMO. In this work, the focus is placed on the transfer process and its application to the BFMO LSC material. Follow‐up works will focus on the functional properties to achieve those necessary for device applications. The magnetic hysteresis loops (M–H) are shown in Figure 1c,d at 300 and 10 K, respectively. While thermal action causes a large change in saturation magnetization and coercivity at low temperatures, the room temperature magnetic properties are still relatively strong. Previous study of the BFMO LSC material has shown that it is a ferrimagnetic material.^[^ ^32^, ^33^, ^34^, ^37^ ^]^ The simultaneous ferroelectricity and ferrimagnetism in BFMO make it an excellent choice for devices such as four‐state memory, spin filters, and photovoltaics.^[^ ^44^, ^45^, ^46^ ^]^

To understand the motivation behind employing the film transfer process to BFMO, direct growth films on LNO and Si substrates were prepared as references. These samples are expected to show very poor‐quality growths as BFMO is not expected to grow well directly on LNO or Si substrates. In both cases, CeO_2_ is used as a buffer layer. The XRD results for each of these samples are shown in Figure 1e,f, respectively. In the BFMO on LNO sample, it is observed that many phases of the film formed on the substrate, including several that cannot be easily identified by their peak positions. This lack of phase purity indicates a poor‐quality growth that is not expected to maintain the desired properties. This is due to the absence of an epitaxial relationship between the LNO lattice (*a*_LNO_ = 5.151 Å) and the CeO_2_ lattice ($a_{{\text{CeO}}_{2}}$ $/\sqrt{2}$ = 3.826 Å, considering 45‐degree rotation), resulting in polycrystalline growth.^[^ ^2^, ^47^ ^]^ In the BFMO on Si, only a single film peak belonging to CeO_2_ is observed in the XRD results, indicating that the BFMO film directly grown on Si was amorphous or had poor crystallinity. Again, the lack of lattice matching between the large Si lattice (*a* = 5.431 Å) and the smaller CeO_2_ lattice (*a* $/\sqrt{2}$ = 3.826 Å) severely limits the growth of the oxide films.^[^ ^2^ ^]^ These results illustrate the difficulty of growing oxide thin films directly on new substrates, such as LNO and Si. While complex buffer layer stacks can be designed to allow films to be grown on substrates with dissimilar lattice parameters, these stacks are time intensive to design and test and only allow growth on one specific substrate.^[^ ^2^, ^48^, ^49^ ^]^ Thin film transfer does not have such limitations as the film can be transferred to virtually any substrate once a high‐quality growth has been achieved with the dissolvable buffer layer.

**Figure 2:**
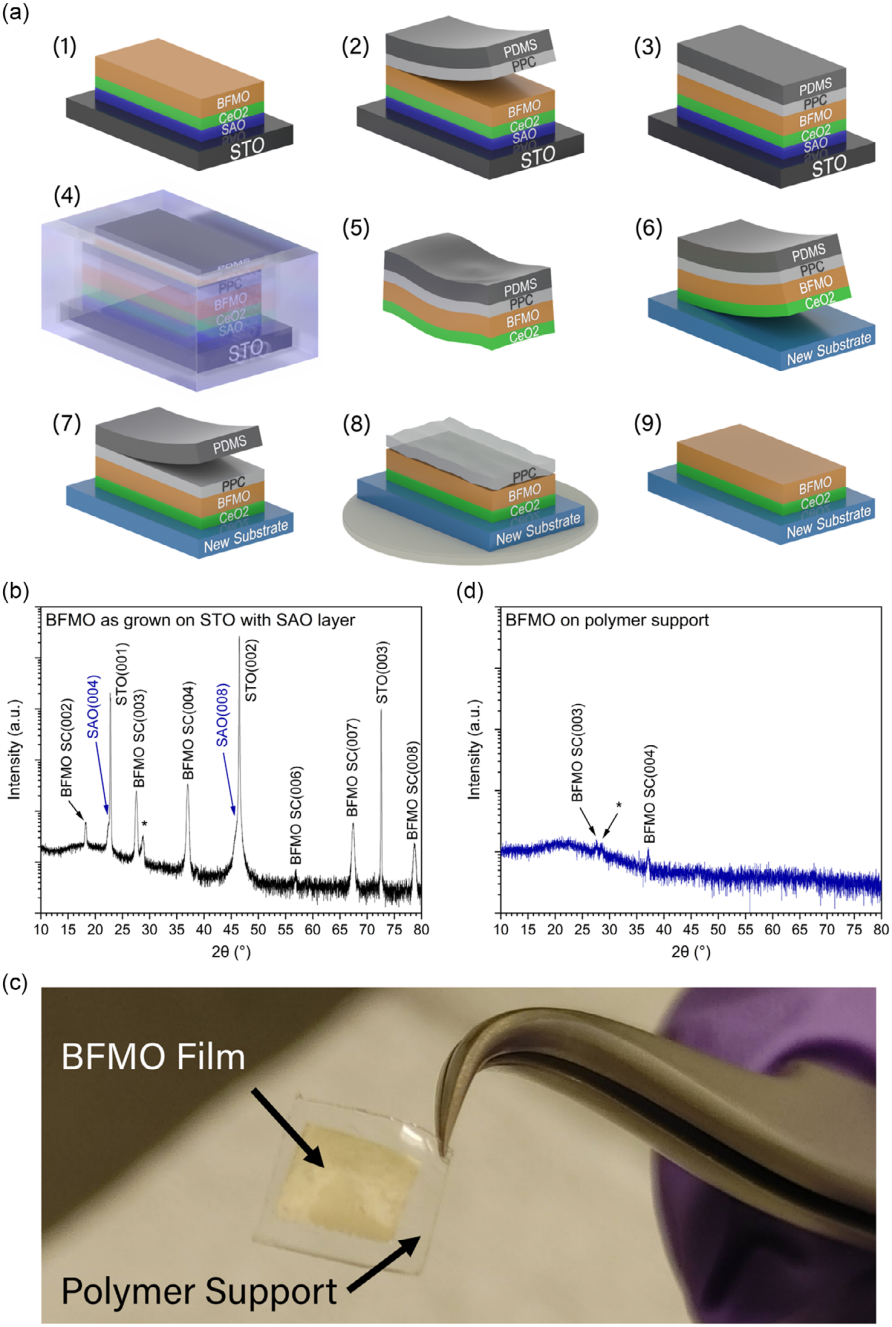
Transfer process for BFMO film using SAO water‐soluble layer with a PDMS/PPC polymer support layer. a) Schematic illustrations of the main steps of the transfer from an STO substrate onto a new substrate. b) XRD results of as‐grown film with SAO layer inserted. c) An optical image of the BFMO film on the polymer support layer after dissolving the SAO and removing the STO substrate. d) XRD on freestanding BFMO film on the polymer support layer after dissolving the SAO but before adhering it to the new substrate. In both XRD scans, peaks marked with the “*” symbol are attributed to a highly strained interlayer at the substrate interface and small grains of varied compositions.

Based on these motivations, a transfer process was designed and optimized for the strained BFMO LSC material. See the thin film transfer methods portion of the experimental section for more specific details on the final, optimized transfer process. The main steps of the transfer process are shown in **Figure** **2**a. 1) The films were deposited on the STO substrate, adding the SAO water‐soluble layer directly on the STO. Figure 2b shows XRD analysis of the film at this point, demonstrating the epitaxial nature of the BFMO film even with the added SAO. The SAO lattice is very similar to that of the STO, explaining the overlapping peaks. 2) The PDMS/PPC flexible polymer bilayer was attached to the surface of the film to act as a support layer during the transfer process. 3) Heat was used to secure the polymer onto the film. 4) The entire sample was placed in a beaker of pure deionized (DI) water to dissolve the SAO layer. 5) After removing the flexible polymer support from the water, the film remained attached. Figure 2c shows a photograph taken at this stage of the transfer process, where the gold‐colored BFMO film is visible on the polymer support. Figure 2d contains the XRD analysis of the mid‐transfer BFMO film on polymer. While several BFMO peaks are still present, the intensity is relatively low. We theorize that there are two reasons for this. First, the transferred film is no longer perfectly flat which posed challenges to accurate XRD measurements. Second, the film quality could be reduced due to the transfer process. Interestingly, this damage appears to be entirely recoverable based on the XRD results on the post‐transfer samples, where recrystallization has taken place during the annealing step at 500 °C. Overall the presence of BFMO LSC peaks indicates that the LSC crystal structure remains in the transferred film. 6) The polymer support was then used to place the film onto the new substrate surface. 7) The PDMS polymer was removed from the sample by peeling. 8) The PPC polymer was evaporated from the sample at elevated temperature and the sample was annealed. We expect that the annealing step will cause recrystallization and address the issue of the low intensity XRD peaks. 9) Finally, the sample was gently cleaned, yielding the BFMO film physically bonded to the new substrate.

**Figure 3:**
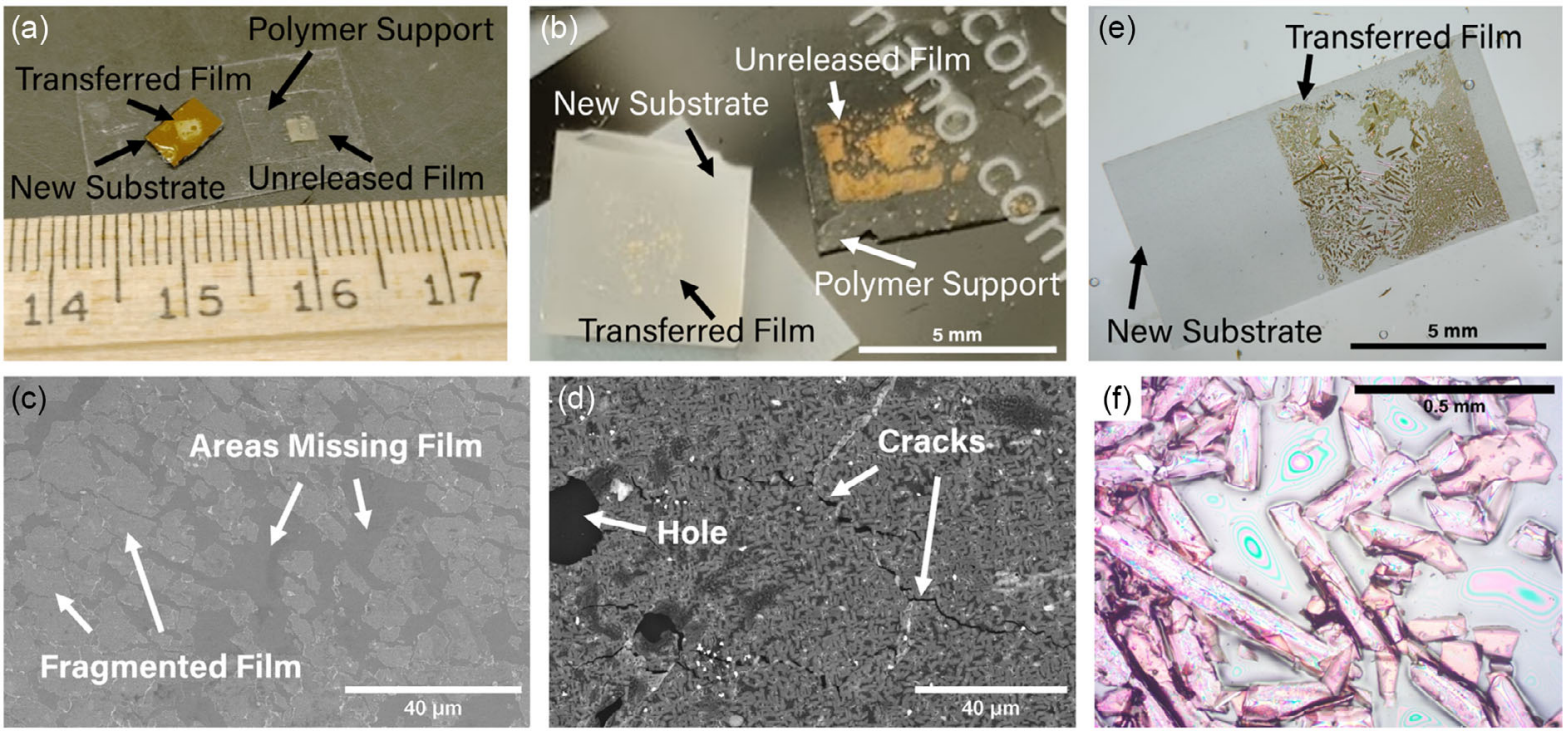
Challenges while optimizing the film transfer process. a,b) Photographs of partially transferred films with areas of film remaining on polymer support layer due to inability to release onto new substrate. c,d) SEM images of corresponding samples with missing and damaged film areas indicated. e) Photograph and f) optical microscopy of sample showing that film curled to release strain.

The steps laid out earlier provide a simple overview of the transfer process, however significant challenges were encountered before a successful transfer was achieved. Most issues were encountered during steps 7–9 (see Figure 2a), where the polymers are supposed to be removed from the sample leaving the transferred film attached to the new substrate. Initially, the transfer was performed using only the PDMS layer—a method that was shown to be successful in other works.^[^ ^7^, ^8^ ^]^ The PDMS material has larger thermal expansion than that of the oxide films, therefore these works have shown the possibility to release the film from the polymer by heating the sample and peeling off the PDMS, since adhesion is lost as the polymer expands relative to the film. Attempts to replicate that process in this work were unsuccessful, with large areas of the transferred film remaining permanently attached to the polymer rather than releasing onto the new substrate. Photographs of such failures are shown in **Figure** **3**a,b, where portions of unreleased film are left on the polymer support layer. Figure 3c,d show scanning electron microscopy (SEM) images of the transferred films, highlighting the poor overall continuity of the transferred films.

Because of these challenges with removing the PDMS polymer layer after transfer, a different polymer is needed to be added to control the surface adhesion and allow the entire film to be left on the new substrate. It was found that this could be achieved by adding a thin layer of PPC to the PDMS before attaching it to the as‐deposited film. This PPC directly contacts the BFMO film while the PDMS acts as a handle and support layer. However, even after adding the PPC layer to improve release, heat alone was insufficient to release the film from the polymer. Subsequently, a different method was studied which involved dissolving the PPC in a solvent, such as acetone, rather than relying on heat to delaminate the film.^[^ ^9^ ^]^ In this process, the PDMS was first peeled away, leaving only the PPC to remove with the solvent. However, this method was unsuccessful and thus was not further pursued because of challenges with the highly strained film, namely the tendency to curl to release strain. Figure 3e shows a photograph of a film after removing the PPC using acetone, where the film shattered and curled into scroll‐like morphologies. Figure 3f shows optical microscopy of the results. Figure S2a–c, Supporting Information, show additional optical microscopy images of films that fragmented into small pieces or folded over, damaging the final film. Figure S3a,b, Supporting Information, show SEM images of additional damaged films with small fragments. Based on these findings, it was determined that using solvents to dissolve the PPC was not a viable option. However, PPC has a low evaporation temperature of 250 °C, making it easy to remove by heating.^[^ ^3^, ^9^ ^]^ Therefore, evaporation of the PPC was instead used as a way of removing it, yielding the superior results reported in this work. The various release approaches have been summarized in Table S1, Supporting Information, with relevant literature references.

Successful large‐area transfers were achieved by peeling the PDMS away and heating the sample to 250 °C to evaporate the PPC, leaving only the BFMO film on the new substrate. A final annealing step was performed at 500 °C to heal defects in the film and establish physical bonding between the film and the new substrate. By using this heating profile, the film surface was also cleaned of any polymer residue. The final annealing step at 500 °C does introduce challenges when working with some of the temperature sensitive systems, such as some polymer substrates, which are unstable at such temperatures. For those temperature sensitive cases, the 500 °C post transfer annealing step could be removed at the expense of some film quality.

**Figure 4:**
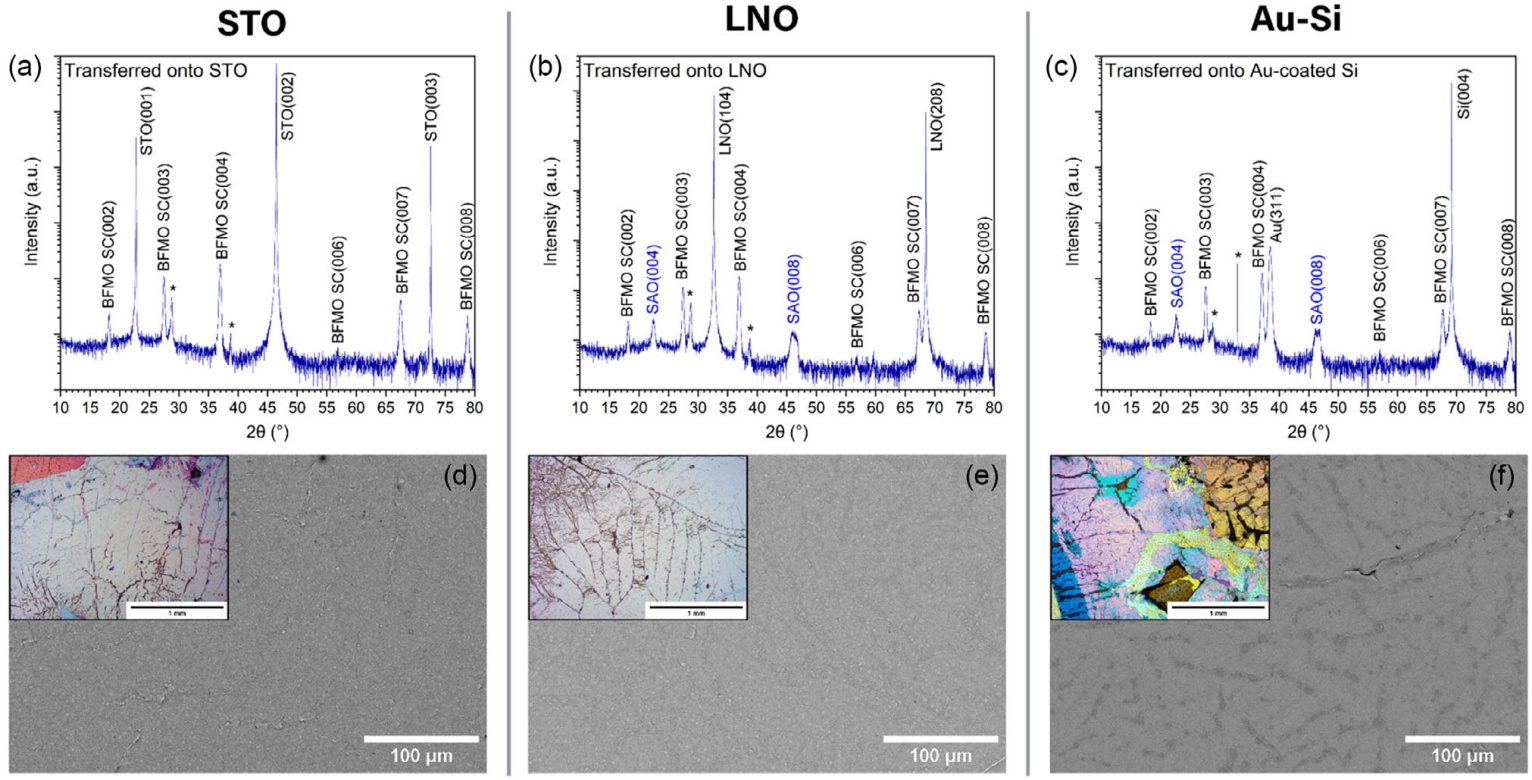
Structural analysis of final transferred films. XRD data of BFMO films transferred onto a) STO, b) LNO, and c) Au‐coated Si. Peaks marked with the “*” symbol are attributed to a highly strained interlayer at the substrate interface and small grains of varied compositions. SEM images of corresponding films on d) STO, e) LNO, and f) Au‐coated Si. Insets show optical microscopy of corresponding films.

Based on the targeted applications, this work focused on transfer of the BFMO film onto substrates including STO, LNO, and Au‐coated Si. The STO (identical to original growth substrate) was used as a baseline to study transfer quality independent of the substrate material. LNO was used to demonstrate potential optical and acoustic applications of the BFMO film, such as acoustically driven ferromagnetic resonance (ADFMR).^[^ ^40^, ^41^ ^]^ The Au‐coated Si was chosen to substantiate potential electronic device applications on silicon and allow for electrical measurements via the bottom electrode of Au. **Figure** **4**a–c show the XRD data for these three final transferred samples. Although the mid‐transfer XRD data shown previously in Figure 2d had poor peak intensity, the final XRD data shows excellent peak intensity. This is attributed to the annealing step that was performed during the transfer process, which caused recrystallization. The high surface roughness of the polymer is also no longer a factor, meaning that X‐ray scattering is not obscuring the crystal peaks. Interestingly, peaks corresponding to SAO are also observed in these XRD scans, albeit at very low intensity. This could either be due to a small portion of the SAO film that did not fully dissolve or it could arise from small grains of the pseudocubic phase of BFMO, which has a similar lattice parameter to that of the SAO film.^[^ ^37^ ^]^ Also notable is the fact that the peak intensity and full‐width at half‐max (FWHM) values are comparable to the original as‐grown XRD data in Figure 2b, indicating that the final transferred film is nearly identical to the as‐grown film on the nanoscale. However, for device purposes it is important that it also maintains its quality on the microscale. To study this further, SEM and optical microscopy were used to image the films at the micro‐ and millimeter scale. Figure 4d–f show the SEM images and include the corresponding optical images as insets. Across the total 5 × 10 mm^2^ area transferred, the SEM reveals continuous, crack‐free BFMO film across several 100 s of microns—surpassing any previous transfer attempts. The optical microscopy images show areas of the film where cracking is worse, establishing the need for further work on the transfer of strained thin films before it could be viable for wafer‐scale applications. Regardless, the scales achieved here mark a considerable step forward in the field. The optical microscopy images give rise to the discovery of another challenge in the transfer process, namely film folding. The variation in color shown in the images indicates that the BFMO film folded on top of itself in some areas. This is especially visible in the Au–Si sample. The Au–Si sample also has more color by nature of the Au coating whereas the STO and LNO substrates are colorless. Figure S2d–f, Supporting Information, show additional higher magnification optical microscopy of the film transferred onto STO. Figure S3c,d, Supporting Information, show additional lower magnification SEM images of these films. An analysis of the XRD, SEM, and optical microscopy images also leads to the conclusion that the post‐transfer sample purity is high as no contaminates can be observed in any of these methods.

**Figure 5:**
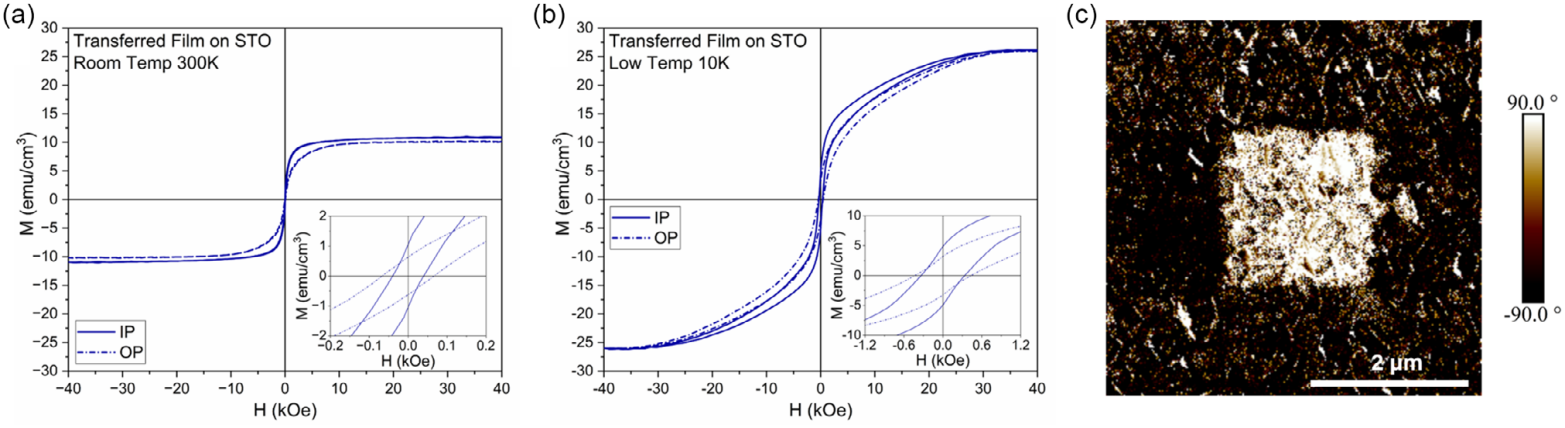
Physical properties of post‐transfer BFMO films. Magnetic properties of BFMO transferred onto STO at a) 300 K and b) 10 K. c) PFM phase map results showing domain switching behavior in BFMO transferred onto Au‐coated Si.

After studying the structure of the transferred BFMO films, it is important to also confirm that the functional properties are still present and have not been compromised after transfer. First, the magnetic properties were studied by performing an M–H measurement on the film transferred onto STO. The film on STO was used to eliminate the substrate as a factor in the measurement comparison. The results are shown in **Figure** **5**a,b at 300 and 10 K, respectively. Interestingly, the saturation magnetization and coercivity values are found to increase post‐transfer. This agrees with the findings of other researchers and is attributed to the release of strain in the film.^[^ ^7^ ^]^ Since the BFMO film is highly strained, even a partial relaxation will have a notable effect on the magnetic properties. The ferroelectric properties of the post‐transfer film were also studied using PFM. The reason that PFM was selected is that the typical capacitor structures used for the P–E measurement were challenging on the current transferred samples due to limited sample areas, as well as defects, pinholes, and cracks in the transferred films. One of the main benefits of PFM is that the AFM probe is used as the top electrode, so no metal contacts are necessary. This allows for ferroelectric measurements even when defects are present. It is expected that the transferred film areas could be made larger with additional optimization of the overall transfer process. The PFM measurement technique uses an AC voltage to read the ferroelectric domain state. After setting the domain state using a DC voltage, retention can be measured by scanning the sample with an AC voltage probe. Figure 5c shows the resulting phase map of the sample surface after writing a domain orientation of 90° in the center box and −90° around the perimeter using opposite DC voltages. The corresponding PFM magnitude image is shown in Figure S4, Supporting Information, where the contrast is low. The observed domain orientation remaining after writing indicates the presence of ferroelectricity in the sample, even though it is very weak. This method was selected because the P–E measurement requires a top electrode and therefore cannot be performed on the small pieces of transferred film. The PFM method only requires ≈5 × 5 μm of continuous film to complete the measurement. The remaining magnetic and ferroelectric properties in the sample indicate a successful transfer and the potential applications of this process.

This transfer method can be incorporated into current oxide thin films on STO substrates and has been shown to preserve the structure and functional properties of the material. Transferring BFMO films onto LNO substrates is significant for acoustic devices^[^ ^40^, ^41^, ^43^ ^]^ and photonics,^[^ ^50^, ^51^ ^]^ which have been limited by the epitaxial growth of films on this substrate. While direct integration of various thin film materials onto LNO and Si substrates has been successfully done, each new film poses a new challenge that takes time and effort to solve.^[^ ^2^ ^]^ As shown in this work, the BFMO LSC material cannot be grown directly on LNO or Si. However, this transfer process allows this film to be transferred onto various substrates without stringent requirements for direct epitaxy. In this work, we have primarily focused on the additional challenges associated with the transfer of strained thin films. By adjusting the transfer methods previously reported in literature, even these highly strained films have been transferred successfully as reported here. However, there is still potential for future work in this area as the films transferred here were ≈ 700 × 700 μm^2^—large enough for individual device fabrication, but not the millimeter‐ or centimeter‐scale that would be desired for commercial wafer‐bonding type applications. Additionally, this method could be applied to many other strained oxide films based on the type of applications needed.

## 3 Conclusions

This work demonstrates the challenges and solutions to the transfer of highly strained BFMO layered oxide thin films. Although examples of thin film transfer can be found in literature, up to this point there has not been a detailed study of the transfer of highly strained oxide thin films. In this work, we have shown that the BFMO LSC phase has magnetic and ferroelectric properties but cannot be grown epitaxially directly on LNO or Si substrates. As a solution, we have demonstrated how BFMO can be grown epitaxially with the SAO water‐soluble layer on the STO substrates. The SAO layer was then dissolved to release the film from the STO substrate and a polymer stack of PPC and PDMS was used to support the film as it was transferred onto the new substrate. A significant challenge that was addressed was the removal of the polymer once the film was transferred. We found that the best results were obtained when the PDMS layer was peeled and the PPC layer was evaporated, leaving the film on the new substrate. With this method, we were able to achieve large continuous film areas of 700 × 700 μm^2^. We also showed that the structure was maintained after transferring onto STO, LNO, and Au–Si as confirmed by XRD, optical microscopy, and SEM. The multiferroic properties that make BFMO an interesting material candidate were also shown to be maintained after transfer, with the magnetic saturation magnetization increasing when compared to the as‐deposited sample due to the strain relaxation. This approach to film transfer is widely applicable and may be used to transfer other oxide films from the growth substrates onto other substrates of practical interest.

## 4 Experimental Section

4.1

4.1.1

##### Thin Film Growth

The films reported in this work were deposited by pulsed laser deposition (PLD) using a KrF excimer laser (*λ* = 248 nm) with a beam incidence angle of 45° and a target‐substrate distance of 4.5 cm. This includes films of Bi_3_Fe_2_Mn_2_O_*x*_, CeO_2_, and Sr_3_Al_2_O_6_, deposited with laser energies of 450, 450, and 420 mJ, respectively, as measured by the laser source. The BFMO target was created by mixing Bi_2_O_3_, Fe_2_O_3_, and MnO_2_ oxide powders together at atomic ratios of Bi:Fe:Mn = 2.1:1:1 (5% excess of Bi), pressing the target in a manual hydraulic press, and sintering in a tube furnace in atmosphere for 3 h at 750 °C. The deposition chamber was pumped to at least the $2\times 10^{-6}$ Torr range before each deposition, at which point 50–200 mTorr of oxygen background pressure was added to aid in the deposition of oxide thin films. The thicknesses of the BFMO and CeO_2_ films were measured to be 150 and 45 nm, respectively. All films were also annealed during substrate cooling (10 °C min^−1^) in a 200 Torr oxygen atmosphere. Direct depositions were performed onto SrTiO_3_(001), LiNbO_3_(104), and Si(001) single‐crystal substrates at temperatures ranging from 700 to 850 °C. For multilayer depositions, all films were deposited sequentially before venting the chamber and unloading the substrate.

##### Microstructure Characterization

XRD (PANalytical Empyrean) with parallel beam optics and a Cu Kα1 monochromator was used to initially characterize the films to confirm the presence of the desired phases and ensure epitaxial quality of the samples. For each sample scanned with XRD, the beam was first aligned to a known substrate peak with the omega, phi, chi, and 2theta axes to maximize the accuracy of the scan. Optical microscopy (Olympus BX41) was performed to analyze the macroscale film quality. SEM (Teneo Volumescope) was used for microscale features. A beam voltage of 10 kV and a current of 50 pA was used for imaging. Characterization of the insulating BFMO film was performed in the low vacuum SEM mode. All SEM images shown in this work were collected with a backscattered electrons (BSE) detector.

##### Property Characterization

The ferroelectric polarization hysteresis measurements were performed using the Radiant Technologies Precision LC II Ferroelectric Tester with a maximum voltage of 1 V and a measurement frequency of 1 Hz. The thin film capacitors used for the ferroelectric P–E measurements on the as‐deposited BFMO films were fabricated using an SrRuO_3_ (SRO) bottom electrode and Au top contacts. The Au contacts were 312 μm in diameter and 150 nm thick and were deposited via DC sputtering through a shadow mask to create a grid of devices for measurement. The magnetization measurements were obtained using the Quantum Design MPMS‐3 SQUID magnetometer in VSM mode. A maximum saturation magnetic field of 40 kOe was applied for all hysteresis loop measurements. Corrections were applied as provided by Quantum Design to account for the dipole assumption made by the SQUID for IP and OP measurements. The samples were cooled in a helium atmosphere at 50 K min^−1^ and allowed to stabilize for 1 min before measurements. The Bruker Dimension Icon was used with SCM‐PIT conductive probes to characterize the two‐dimensional ferroelectric response via piezoelectric force microscopy (PFM). A DC bias of 3 V was used to “write” the domain orientations before reading them back with an AC bias of 2 V and a frequency of 300 kHz. The transferred films used for the PFM measurements were transferred onto Au‐coated Si to provide a bottom electrode for the ferroelectric measurement. The AFM probe tip acts as the top electrode in this technique.

##### Thin Film Transfer Methods

The water‐soluble Sr_3_Al_2_O_6_ (SAO) layer was deposited along with the other films during the PLD process. Since the deposition was performed under an oxygen atmosphere after pumping to high vacuum, no significant water vapor is present during the deposition. The polymer support layer was fabricated by spin coating three layers of polypropylene carbonate (PPC) onto a poly(dimethylsiloxane) (PDMS) stamp to achieve the desired adhesion with the film. The PPC solution was prepared by mixing 1.5 g of PPC into 10 mL of anisole (15 wt% PPC) and heating at 60 °C for 5 h while stirring the solution. Spin coating was done on a glass side at 1000 rpm for 120 s to form each of the three layers of PPC. The polymer stack was heated to 80 °C for 3 min after each spin to partially crosslink the PPC and increase the viscosity. After the final cool to room temperature, the PPC was cut around the edges to release the stack from the glass slide. The flexible polymer stack was then attached to the cleaned film surface (with the PPC side contacting the film) and heated at 70 °C intermittently to establish adhesion to the film as air bubbles were pushed out. A final bake of 70 °C for 2 min was used to secure the polymer to the film. The entire substrate‐film‐polymer stack was placed in deionized (DI) water overnight to etch the SAO layer. The polymer was then gently pulled from the substrate, removing the film with it, and dried in a desiccator overnight. The film‐polymer stack was then placed onto the cleaned surface of the new substrate and heated at 80 °C for 3 min while pressing down with a flat object to force air out from between the film and the new substrate. The PDMS was then peeled away, leaving the PPC and film on the new substrate. The sample was then heated at 80 °C for 5 min to allow the PPC to soften and conform to the new substrate to secure the film down. Finally, the sample was placed in a tube furnace and annealed at 500 °C for 3 h (plus a 1 h hold at 250 °C; heating and cooling at 5 °C min^−1^) to evaporate the PPC and reestablish the chemical bonding between the new substrate and the film.

## Conflict of Interest

The authors declare no conflict of interest.

## Supporting information

Supplementary Material

## Data Availability

The data that support the findings of this study are available in the supplementary material of this article.
