# Supplementary material for: Large Area Transfer of Bismuth‐Based Layered Oxide Thin Films Using a Flexible Polymer Transfer Method
Source: Small Sci. 2024 Jun 30;4(9):2400114. doi: 10.1002/smsc.202400114 (PMC11935224; doi:10.1002/smsc.202400114)
Supplement: Supplementary file 1 — Supplementary Material [file SMSC-4-2400114-s001.pdf]

Large Area Transfer of Bismuth-Based Layered Oxide Thin Films using a Flexible Polymer Transfer Method

James P. Barnard,[1] Jianan Shen,[1] Benson Kunhung Tsai,[1] Yizhi Zhang,[1] Max R. Chhabra,[1] Ke Xu,[1] Xinghang Zhang,[1] Raktim Sarma,[2,3] Aleem Siddiqui,[2] Haiyan Wang[1,4,*]

[1]*School of Materials Engineering, Purdue University, West Lafayette, IN 47907, USA*
[2]*Sandia National Laboratories, Albuquerque, NM, 87185, USA*
[3]*Center for Integrated Nanotechnologies, Sandia National Laboratories, Albuquerque, NM, 87185, USA*
[4]*School of Electrical and Computer Engineering, Purdue University, West Lafayette, IN 47907, USA*
[*]*Corresponding Author E-mail: hwang00@purdue.edu*

Supporting Information:

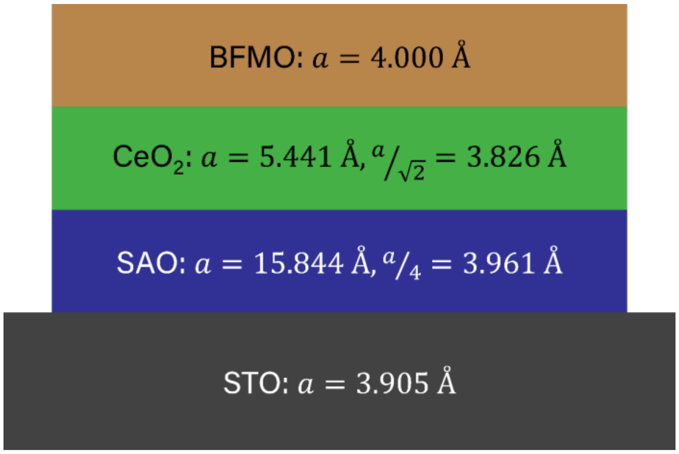

Figure S1. Schematic illustration of the epitaxial lattice matching of the various layers of the stack. This is important as epitaxial growth has been achieved even with the added SAO water-soluble layer.

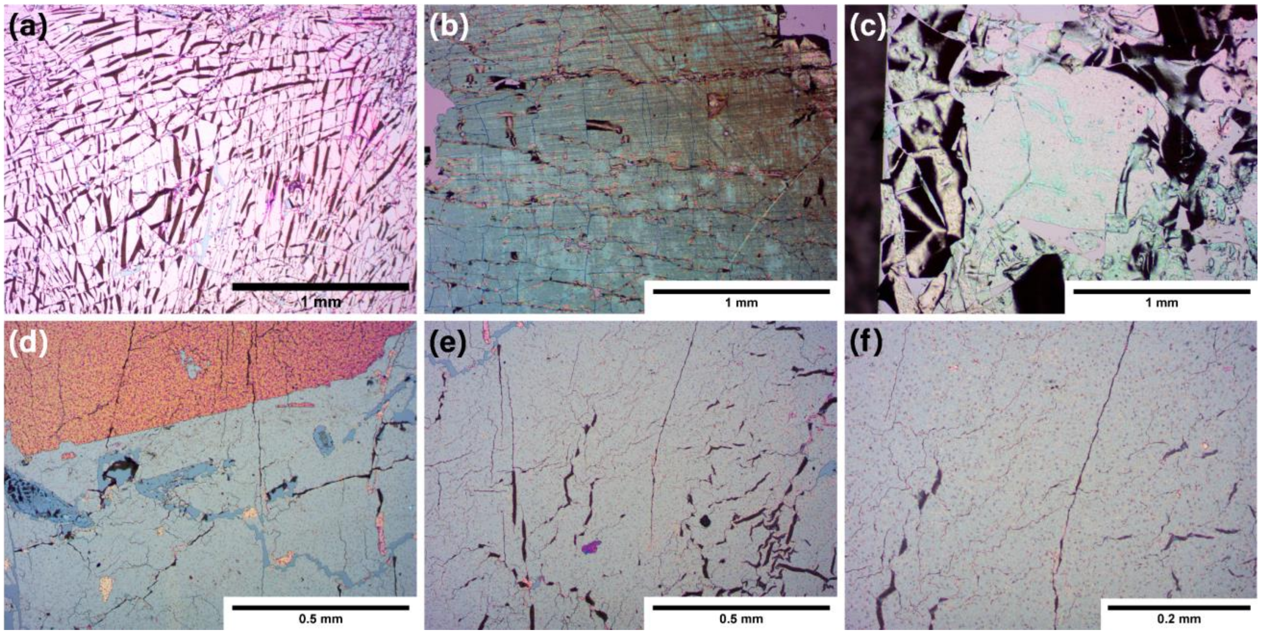

Figure S2. Optical microscopy of post-transfer films on STO. (a) Film broken into small fragments. (b) Improved transfer area, but poor film continuity. (c) Film folded over on top of itself. (d-f) Higher magnification images of final successfully transferred film.

Table S1. Summary of reported film release methods.

Name of Method	Description of Method	References
PDMS Peeled Only	Only one polymer is used for the support, PDMS. After attaching the film to the new substrate, the PDMS is peeled away, leaving the film on the new substrate.	[1–11]
PDMS Peeled + PPC Dissolved	Both PDMS and PPC polymers are used for support. After the film is attached to the new substrate, the PDMS is first peeled off. Second, the PPC is dissolved in acetone or another solvent, leaving the film on the new substrate.	[12–15]
PDMS Peeled + PPC Evaporated	Both PDMS and PPC polymers are used for support. After the film is attached to the new substrate, the PDMS is first peeled off. Second, the PPC is evaporated at 250 °C, leaving the film on the new substrate.	[13]

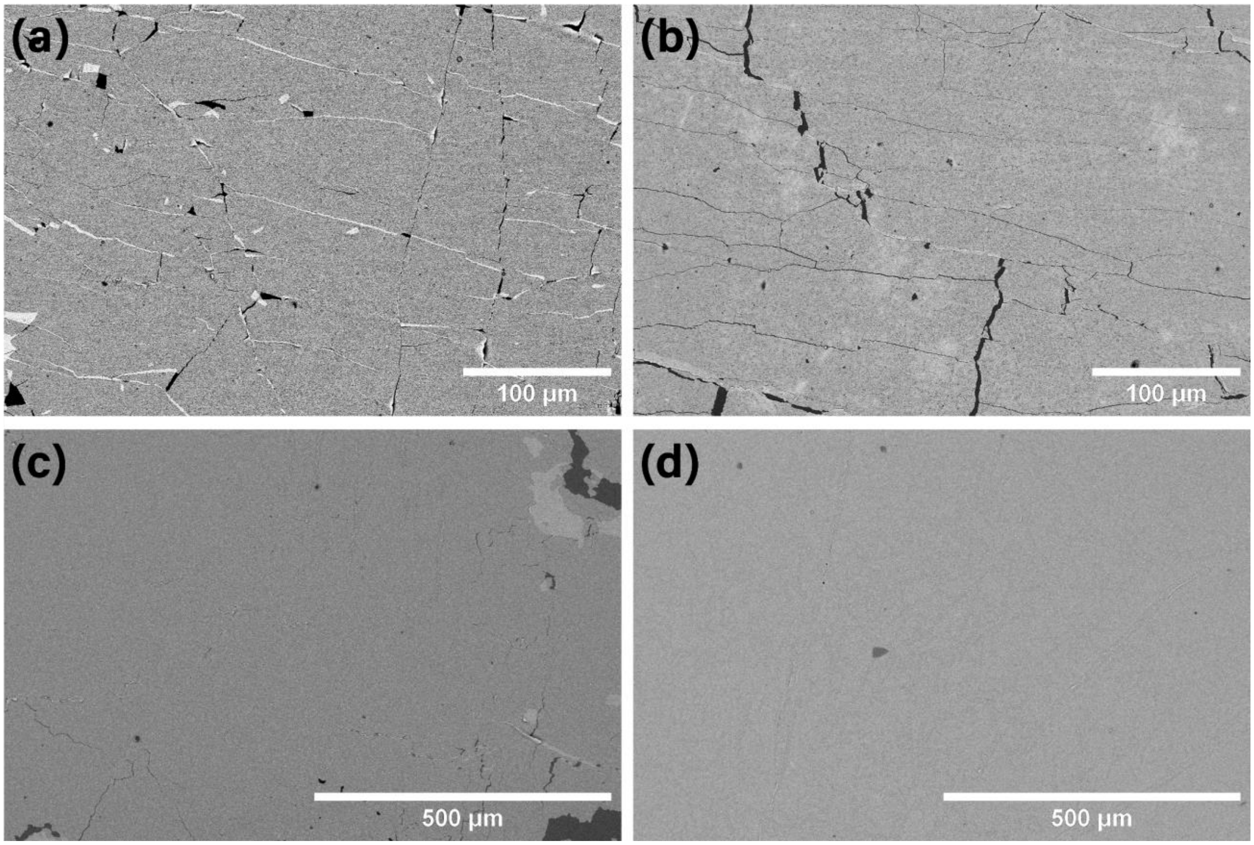

Figure S3. SEM images of post-transfer films. (a, b) Films transferred onto STO with poor film continuity and many small fragments. Lower magnification images of final successfully transferred films on (c) STO and (d) LNO.

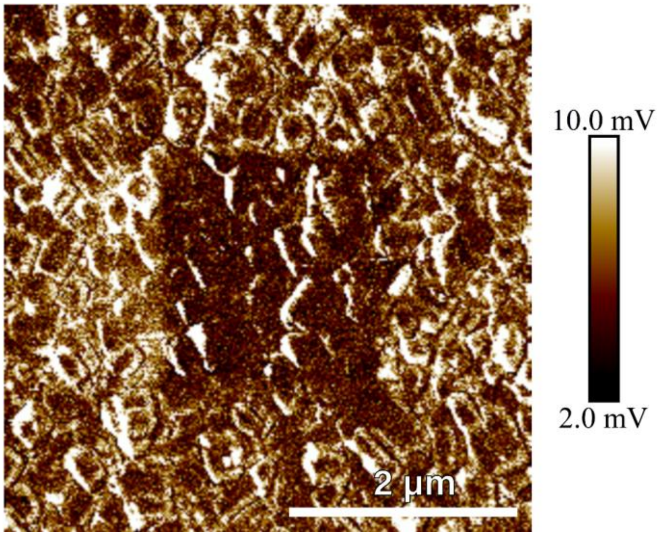

Figure S4. PFM magnitude map of final transferred film on Au-coated Si. Area matches that of the phase map.

References:

[1] B. Peng, R.-C. Peng, Y.-Q. Zhang, G. Dong, Z. Zhou, Y. Zhou, T. Li, Z. Liu, Z. Luo, S. Wang, Y. Xia, R. Qiu, X. Cheng, F. Xue, Z. Hu, W. Ren, Z.-G. Ye, L.-Q. Chen, Z. Shan, T. Min, M. Liu, *Sci Adv* **2020**, *6*, DOI 10.1126/sciadv.aba5847.

[2] G. Dong, Y. Hu, C. Guo, H. Wu, H. Liu, R. Peng, D. Xian, Q. Mao, Y. Dong, Y. Zhao, B. Peng, Z. Wang, Z. Hu, J. Zhang, X. Wang, J. Hong, Z. Luo, W. Ren, Z. Ye, Z. Jiang, Z. Zhou, H. Huang, Y. Peng, M. Liu, *Advanced Materials* **2022**, *34*, DOI 10.1002/adma.202108419.

[3] G. Dong, S. Li, M. Yao, Z. Zhou, Y.-Q. Zhang, X. Han, Z. Luo, J. Yao, B. Peng, Z. Hu, H. Huang, T. Jia, J. Li, W. Ren, Z.-G. Ye, X. Ding, J. Sun, C.-W. Nan, L.-Q. Chen, J. Li, M. Liu, *Science (1979)* **2019**, *366*, 475.

[4] F. An, K. Qu, G. Zhong, Y. Dong, W. Ming, M. Zi, Z. Liu, Y. Wang, B. Qi, Z. Ding, J. Xu, Z. Luo, X. Gao, S. Xie, P. Gao, J. Li, *Adv Funct Mater* **2020**, *30*, DOI 10.1002/adfm.202003495.

[5] Z. Zhao, A. Abdelsamie, R. Guo, S. Shi, J. Zhao, W. Lin, K. Sun, J. Wang, J. Wang, X. Yan, J. Chen, *Nano Res* **2022**, *15*, 2682.

[6] P. Salles, I. Caño, R. Guzman, C. Dore, A. Mihi, W. Zhou, M. Coll, *Adv Mater Interfaces* **2021**, *8*, DOI 10.1002/admi.202001643.

[7] A. Jain, P. Bharadwaj, S. Heeg, M. Parzefall, T. Taniguchi, K. Watanabe, L. Novotny, *Nanotechnology* **2018**, *29*, 265203.

[8] Y. Kim, S. S. Cruz, K. Lee, B. O. Alawode, C. Choi, Y. Song, J. M. Johnson, C. Heidelberger, W. Kong, S. Choi, K. Qiao, I. Almansouri, E. A. Fitzgerald, J. Kong, A. M. Kolpak, J. Hwang, J. Kim, *Nature* **2017**, *544*, 340.

[9] D. Lu, D. J. Baek, S. S. Hong, L. F. Kourkoutis, Y. Hikita, H. Y. Hwang, *Nat Mater* **2016**, *15*, 1255.

[10] J. Zhang, T. Lin, A. Wang, X. Wang, Q. He, H. Ye, J. Lu, Q. Wang, Z. Liang, F. Jin, S. Chen, M. Fan, E.-J. Guo, Q. Zhang, L. Gu, Z. Luo, L. Si, W. Wu, L. Wang, *Science (1979)* **2024**, *383*, 388.

[11] J. Shen, B. K. Tsai, K. Xu, A. Shang, J. P. Barnard, Y. Zhang, R. Tripathi, Z. Chen, X. Zhang, H. Wang, *Nano Res* **2023**, *16*, 10559.

[12] F. Pizzocchero, L. Gammelgaard, B. S. Jessen, J. M. Caridad, L. Wang, J. Hone, P. Bøggild, T. J. Booth, *Nat Commun* **2016**, *7*, 11894.

[13] D. Pesquera, E. Parsonnet, A. Qualls, R. Xu, A. J. Gubser, J. Kim, Y. Jiang, G. Velarde, Y. Huang, H. Y. Hwang, R. Ramesh, L. W. Martin, *Advanced Materials* **2020**, *32*, DOI 10.1002/adma.202003780.

[14] D. G. Purdie, N. M. Pugno, T. Taniguchi, K. Watanabe, A. C. Ferrari, A. Lombardo, *Nat Commun* **2018**, *9*, 5387.

[15] C. M. Went, J. Wong, P. R. Jahelka, M. Kelzenberg, S. Biswas, M. S. Hunt, A. Carbone, H. A. Atwater, *Sci Adv* **2019**, *5*, DOI 10.1126/sciadv.aax6061.
